# Introducing hepatitis C virus healthcare pathways in addiction care in the Netherlands with a Breakthrough project: a mixed method study

**DOI:** 10.1186/s12954-019-0316-4

**Published:** 2019-07-15

**Authors:** Patricia A. M. Kracht, Elisabeth A. de Gee, Agnes van der Poel, Marc A. M. T. Verhagen, Andy I. M. Hoepelman, Esther A. Croes, Joop E. Arends

**Affiliations:** 1 Department of Internal Medicine and Infectious Diseases, University Medical Center Utrecht, Utrecht University, Utrecht, the Netherlands; 2 Netherlands Institute of Mental Health and Addiction (Trimbos Institute), Utrecht, the Netherlands; 3 Department of Gastroenterology and Hepatology, Diakonessen Hospital, Utrecht, the Netherlands

**Keywords:** Cascade of referral, Hepatitis C virus, Addiction care, People who inject drugs, Breakthrough project, Quality Improvement Collaborative, Micro-elimination

## Abstract

**Background:**

People who inject drugs (PWID) are disproportionally affected by the hepatitis C virus (HCV) infection. In the Netherlands, active HCV transmission in PWID has practically been halted but uptake of HCV testing and linkage to care remains insufficient in this risk group. A national HCV in Addiction Care (HAC) quality improvement project based on the Breakthrough methodology (i.e. Breakthrough project) aimed to secure proper linkage to care in PWID by introducing local HCV healthcare screening and treatment pathways in addiction care units.

**Aim:**

To qualitatively appraise the local HCV healthcare pathways; to evaluate the yield in terms of number of PWID screened, diagnosed, referred, and treated; and to identify best practices and barriers to successful participation in the HAC Breakthrough project.

**Methods:**

Between 2013 and 2016, 12 units of addiction care centers throughout the Netherlands participated in two series of a HAC Breakthrough project. Local multidisciplinary teams created HCV healthcare pathways. Quality assessment of HCV healthcare pathways was performed retrospectively and data on screening results was collected. In-depth interviews were conducted to elucidate best practices and essential elements for successful participation.

**Results:**

In total, six HCV healthcare pathways were submitted by ten teams of which 83% was judged to be of “good” or “sufficient” quality. Uptake of HCV-antibody screening was 40% (*N* = 487/1219) and uptake of HCV-RNA in HCV-antibody positives was 59% (*N* = 107/181). The project resulted in 76 (6%) newly detected cases of persistent HCV viremia. Of all HCV-RNA positives, 92% was referred to a hepatitis treatment center. In 39% (N = 27/70) of those referred, treatment initiation was documented and 82% (N = 22/27) achieved a sustained virological response. Teams identified several best practices including motivational counseling training, oral swabs for anti-HCV testing, facilitating complementary HCV RNA testing, and supervised hospital visits.

**Conclusion:**

The HAC Breakthrough project has brought about good quality HCV healthcare pathways in the majority of participating addiction care centers and has successfully linked PWID with ongoing HCV viremia to care. Uptake of HCV screening and treatment after referral were identified as the main gaps to be closed in the HCV cascade of care to achieve final HCV elimination in Dutch PWID (i.e. micro-elimination).

## Introduction

In the Netherlands, the epidemic of heroin use during the 1970s contributed to the creation of a cohort of about 28,000 *substance* users in the early 1990s and up to 89% of those reported to have ever injected drugs [1–3]. In this period, a widespread (global) parenteral transmission of non-A, non-B hepatitis was also observed and in 1989 the hepatitis C virus (HCV) was identified as the culprit [4, 5]. As a result, people who inject drugs (PWID) are disproportionally affected by this predominantly blood-borne infectious disease [6]. This is illustrated by the high prevalence of HCV-antibodies among Dutch PWID, which is estimated to range between 26 and 74% [7–11]. Although the proportion of PWID with a persistent chronic HCV infection appears to have decreased over time from 31% in 2005 to 18% in 2015, as reported by the Amsterdam Cohort Studies, it remains substantial [11]. At the moment, Dutch PWID are confronted with the long-term consequences of chronic HCV which causes cirrhosis (16% over a period of 20 years) but also hepatocellular carcinoma (1–3% per year after 30 years) [12, 13].

Over the past decades, “harm reduction” has gradually become one of the main treatment paradigms for substance use disorders (SUD) in addiction healthcare in the Netherlands. Partly due to such harm reduction practices, including needle-exchange programs, methadone maintenance therapy, and the use of prescription heroin in a controlled setting [14, 15], ongoing HCV transmission in the PWID high-risk group has practically been reduced to zero [16]. This low transmission rate can further be explained by the overall decreasing popularity of injecting drug use which has diminished from 12% (+/−1616 of 13,468 individuals) in 2006 to a historically low proportion of 8.1% (+/−740 of 9093 individuals) of all nationally registered individuals with an opiate use disorder in addiction care in 2015 [17].

The specific subpopulation of people with an opiate use disorder who attend addiction care with high HCV prevalence, negligible transmission, and regular contact with healthcare providers constitutes a well-defined high-risk group that could greatly benefit from targeted HCV micro-elimination efforts, even in the pre-direct-acting antiviral (DAA) period when the project described in this paper started. Unfortunately, even though the Dutch guidelines on opiate maintenance treatment (RIOB) recommend systematic screening for infectious diseases, HCV testing is not routinely facilitated in Dutch addiction care [18]. In addition, the uptake of HCV testing in addiction clinics is still insufficient (53–66%) [7]. The most recent and targeted HCV information campaign reached a linkage to care rate of 77% in opioid substitution clinics (OSTs). However, many organizational barriers for proper and structural linkage to care were identified in this project [19]. Due to local differences in the organization of (HCV) care in addiction clinics, a “bottom-up” approach was deemed the most feasible method to implement improvements. For this reason, the Trimbos Institute (the National Institute on Mental Health and Addiction) initiated a national implementation project based on the Breakthrough method in order to develop local sustainable HCV referral cascades that adequately safeguard linkage to care from addiction care centers to hospitals. The Breakthrough method is an implementation model developed by *The Institute for Healthcare Improvement* in Boston. This method specifically focuses on organizational issues in healthcare systems that may prevent healthcare professionals from complying with current evidence-based practice guidelines [20]. It has been adopted in various healthcare improvement projects all over the world but not to address HCV-related topics in an addiction care setting [21, 22].

## Study aim

The main aim of the Dutch HCV in Addiction Care (HAC) Breakthrough project was to achieve comprehensive and clearly defined local HCV healthcare pathways that are firmly integrated in daily practice in at least 80% of the participating addiction care units and hospitals. With the local HCV healthcare pathways, the HAC Breakthrough project intends to secure proper linkage to (hospital) care of PWID who are diagnosed (in addiction care) with HCV. This study assessed (1) the quality of the implemented healthcare pathways and (2) the yield of the HCV healthcare pathways in terms of number of clients screened and HCV cases detected, referred, and treated. This will be compared with the period before the Breakthrough project; (3) best practices that prevent dropout in the HCV cascade of care and essential elements for successful participation in the Breakthrough project.

## Methods

### HCV in Addiction Care Breakthrough project

**Fig. 1:**
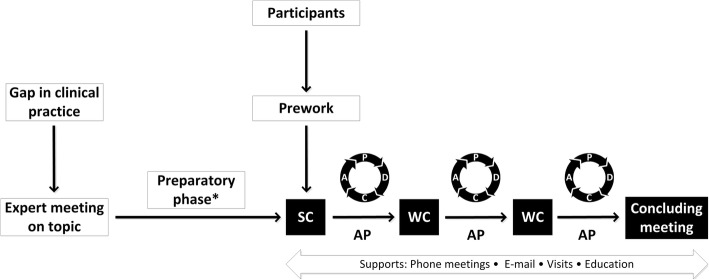
Hepatitis C in addiction care Breakthrough method. Adapted from the Breakthrough Series: IHI’s Collaborative Model for Achieving Breakthrough Improvement. IHI Innovation Series white paper. Boston: Institute for Healthcare Improvement; 2003. (Available on www. IHI.org). SC starting conference, WC working conference, AP action period, PDCA Plan-Do-Check-Act cycle. *Local intake at addiction care units to identify barriers. Management consultation to optimize conditions in terms of available fulltime equivalent. Compilation of project maps & supporting materials

The Breakthrough method was the implementation model of choice due to its ability to achieve substantial healthcare improvements in a relatively short period of time (approximately 9 to 18 months) [20]. This method has frequently been adopted to facilitate the diffusion of existing knowledge into the daily clinical routine [21, 23, 24]. First of all, a “gap” between evidenced-based practices and clinical care is identified. Subsequently, multidisciplinary teams, all involved in the topic of interest, are formed. These so-called Breakthrough collaboratives repeatedly implement small-scale interventions to improve the quality of care and regularly measure change by using a continuous quality improving technique: the “Plan-Do-Check-Act” (PDCA) cycle. In this manner, healthcare interventions can be further refined and finally optimized. During this process, participating teams are educated and coached by experts on the topic of interest and on the Breakthrough method. Also, they attend centrally coordinated conferences that bring together all teams to exchange ideas and experiences. A detailed description of the HAC Breakthrough method is depicted in Fig. 1.

Between 2013 and 2016, two series of the HAC Breakthrough project were initiated by the Trimbos Institute. The incentive for this project had been the evident gap between existing guidelines on HCV care in people who have ever injected drugs and current clinical practice in addiction healthcare that had been identified during a previous HCV information campaign [19]. The HAC Breakthrough project aimed to close this gap by implementing joined HCV healthcare pathways in addiction care centers and the nearby hepatitis treatment centers. The Trimbos Institute launched and coordinated both projects. The expert team consisted of experts on the Breakthrough method and experts on HCV care for PWID.

A total of 12 local care units throughout the Netherlands (representing eight addiction care centers) participated in the two series of the HAC Breakthrough project (both lasted between 1 and 1.5 years). Addiction care centers willing to participate in the Breakthrough project were recruited through the government funded Dutch Network of Infectious diseases & Harm Reduction. Local care units willing to participate were required to obtain approval and support from their management boards prior to final inclusion in the HAC Breakthrough project. All 12 care units formed their own Breakthrough team consisting of at least an addiction specialist, a hepatology specialist, a member of the addiction care management staff, and specialized nurses in both addiction care and hepatology. The multidisciplinary teams were encouraged to create sustainable and systematic HCV healthcare pathways to guide the future HCV screening and referral of PWID. During both series of the HAC Breakthrough project, all teams, including the expert team, gathered at four comprehensive central conferences (i.e., one starting, two working, and one concluding conference). The teams implemented healthcare interventions during the “action periods” that followed the central conferences. By the repeated reassessment of all incorporated steps and/or changes in the HCV healthcare pathway at the central conferences, the implementation of improvements took place in a cyclical PDCA fashion.

### Quantitative data collection

To support the HCV healthcare pathway implementation process, local teams were requested to monitor the local progress from HCV screening towards treatment to this, and a designated database was created which included an algorithm to automatically delete all patient identification data (e.g., name, file number, date of birth) after the data collection had ended. This database included information on age, sex, HBV and human immunodeficiency virus (HIV) status, anti-HCV and HCV-RNA results, HCV genotype, hospital referral, HCV treatment, and sustained virological response (SVR, defined as negative serum HCV RNA 12 weeks post-treatment) status. In this manuscript, available HCV test results for each individual step of the HCV cascade of care are described. Because of the high level of missing data on the HBV and HIV status, these variables were excluded from the analyses. Retrospectively, HCV test results from both before and during the HAC Breakthrough period were compared. Lastly, each team was asked to present their final HCV healthcare pathway at the concluding central conference.

The HAC Breakthrough project was not subject to the Medical Research Involving Human Subjects Act (WMO). Only available data related to clinical care was anonymously collected; therefore, no ethical approval was sought.

### Scoring of HCV healthcare pathways

Quality assessment of all HCV healthcare pathways was performed retrospectively and was based on a 10-item scoring system from the guidebook on healthcare pathways which is used in mental healthcare institutions in the Netherlands (GGZ) [25]. This scoring system included six criteria from Huiskes et al. [26] and was complemented with another four criteria of the European Pathway Association (EPA) [27, 28]. Since these criteria were put together after the finalization of the two projects, teams were not informed on these criteria during the drafting of the pathways. The quality of the HCV healthcare pathways was classified as “Excellent” (score 9 or 10/10), “Good” (score 7 or 8/10), “Sufficient” (score 5 or 6/10), “Insufficient” (score 3 or 4/10), or “Poor” (score 1 or 2/10). Scoring of the HCV healthcare pathways was performed independently and by two professionals of the Trimbos Institute (EG and AP). In the case of different scoring results (which were few), consensus was sought through discussion.

### Statistical analysis of client data

Continuous data are reported as mean with standard deviations (SD) and discrete variables are described as absolute and relative frequencies. Both the non-missing as well as the missing descriptive count data are reported. Differences between subgroups were tested for statistical significance by a complete case analysis with the independent *t* test, Pearson chi-square test, or Fisher’s exact test, as appropriate. A two-sided *p* value < 0.05 was deemed statistically significant for all analyses. SPSS version 24.0 (IBM, Armonk, New York, USA) and GraphPad Prism version 6.0 (GraphPad Software, La Jolla California USA) were used for statistical analyses and creating graphics, respectively.

### Interviews and conference summary reports

Face-to-face semi-structured in-depth interviews were conducted individually with three addiction care members (two nurses and one nurse policy adviser) of different Breakthrough teams to identify best practices and main barriers for successful participation in the HAC Breakthrough project. Interview duration varied between 45 and 60 min. Interviews were transcribed verbatim and entered into the software program NVivo V.11 (NVivo qualitative data analysis Software; QSR International Pty Ltd. Version 11, [29]) for thematic content analysis which was performed by two researchers (PK and AP).

Another source from which best practices were extracted comprised a comprehensive summary of the most successful interventions and contributing factors that were discussed during the central conferences. The summary was drafted by the HAC Breakthrough team of the Trimbos Institute who attended the conferences.

## Results

### HCV healthcare pathways

At the concluding central conference, 10/12 (83%) Breakthrough teams presented their final HCV healthcare pathway. The remaining two teams did not submit their end product to the Trimbos Institute, since they did not succeed to finalize a final draft of their pathway within the timespan of the project. Different teams from the same addiction care center ended up creating a mutual pathway. This resulted in ten teams submitting a total of six HCV healthcare pathways. The quality assessments of the six finalized HCV healthcare pathways are delineated in Table 1. None of the appraised pathways achieved a perfect score by meeting all ten quality requirements. In total, 5 out of 6 (83%) were judged to be of either “good” or “sufficient” quality. One HCV pathway was considered below standard and was scored as “insufficient.” A specification of the “aspired lead time of the steps in the pathways” and also the “monitoring of the compliance of healthcare professionals with pathway steps” was not included in any pathway.

**Table 1:** Scoring of HCV healthcare pathways

Criteria												
Pathways	No. of teams involved	1) Target disease and pathway described?	2) Reference to evidence base?	3) Roles and tasks described?	4) Patient involvement specified?	5) Goal defined?	6) Care process in sequential activities and key decision stages indicated?	7) Deviations from pathway evaluated?	8) Resources identified?	9) Lead time steps characterized?	10) Monitoring of compliance?	Final HCV pathway scoring
1	2	Yes	Yes	Yes	Yes	Yes	Yes	Yes	Yes	No	No	Good
2	2	Yes	No	No	Yes	Yes	Yes	Yes	Yes	No	No	Sufficient
3	1	Yes	Yes	No	Yes	Yes	Yes	No	Yes	No	No	Sufficient
4	1	Yes	No	Yes	No	No	Yes	Yes	Yes	No	No	Sufficient
5	3	Yes	Yes	Yes	Yes	Yes	Yes	No	Yes	No	No	Good
6	1	Yes	No	Yes	No	Yes	No	No	No	No	No	Insufficient

The scoring system is based on the guidebook on healthcare pathways [23] and included six criteria from Huiskes et al. [24] and was complemented with another four criteria of the European Pathway Association (EPA) [25, 26]

### HCV screening results

At the end of the HAC Breakthrough project, 10/12 teams submitted their database with information on the local screening progress, hospital referral, HCV treatment, and SVR, with a total of 1.219 registered clients. These PWID were predominantly male (79%) with a mean age of 50 years (± 9.7). Not all variables were documented completely for all clients.

**Fig. 2:**
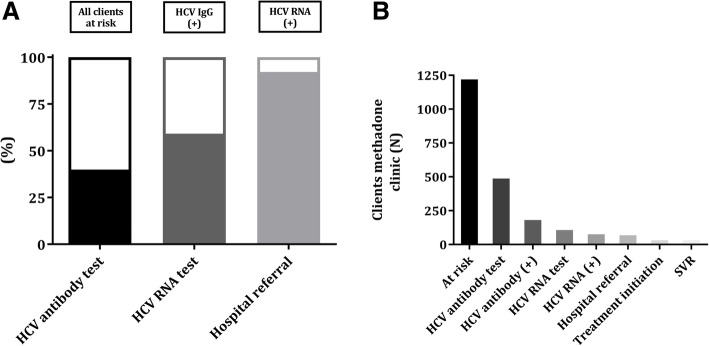
HCV cascade of referral in 10 addiction care units after the Breakthrough project. **a** Completion of each step in the referral cascade in relative frequencies. First bar: percentage of HCV antibody uptake in all methadone clinic clients. Second bar: percentage of HCV RNA uptake in all methadone clinic clients with positive HCV antibodies. Third bar: percentage of hospital referral in all methadone clinic clients with positive HCV RNA. **b** Completion of each step in the referral cascade in absolute numbers. HCV hepatitis C virus, IgG immunoglobulin G, RNA ribonucleic acid, SVR sustained virological response

In total, 40% (*N* = 487/1219) received HCV-antibody testing (Fig. 2) of which 37% (*N* = 181/487) were positive. HCV-antibody testing was performed relatively more often during the HAC Breakthrough period (6.1% each year between 2013 and 2016) than before (2.1% each year between 2002 and 2012) while in 47% the date of screening was unknown.

Subsequent HCV-RNA testing took place in 59% (*N* = 107/181) of the HCV-antibody positives and 71% (*N* = 76/107) of all HCV-RNA diagnostics were positive (Fig. 2). The genotype distribution (as recorded by the addiction care) was as follows: 1 (20%), 2 (5%), 3 (11%), 4 (5%), and missing in 59% (*N* = 76).

Finally, 92% (*N* = 70/76) of the viraemic patients was referred to a nearby hepatitis treatment center and of the 39% (*N* = 27/70) patients with a registered treatment initiation, 82% (*N* = 22/27) achieved a SVR. A summary of all screening results per individual team is delineated in Table 2.

**Table 2:** HCV cascade of referral results in the individual teams

ID	***N***	Age *mean (SD)*	Male gender *(%)*	HCV antibody tested, *n* (%)	HCV antibody (+) in HCV antibody tested, n (%)	HCV RNA tested in HCV antibody (+), *n* (%)	HCV RNA (+) in HCV RNA tested, *n* (%)	HCV RNA (+) referred to hospital, *n* (%)
1.	380	46.8 (10.9)	75	110 (29)	13 (12)	10 (77)	10 (100)	9 (90)
2.	75	47.3 (8.1)	80	25 (33)	7 (28)	3 (43)	3 (100)	2 (67)
3.	134	50.9 (8.8)	84	61 (46)	21 (34)	15 (71)	10 (67)	9 (90)
4.	25	51.9 (8.8)	72	25 (100)	14 (56)	13 (93)	10 (77)	9 (90)
5.	115	49.4 (8.2)	84	64 (56)	32 (50)	18 (56)	11 (61)	11 (100)
6.	61	50.0 (7.4)	80	47 (77)	14 (30)	11 (79)	9 (82)	9 (100)
7.	119	52.0 (6.2)	77	71 (60)	47 (66)	8 (17)	7 (88)	7 (100)
8.	88	50.1 (9.5)	77	27 (31)	8 (30)	7 (88)	4 (57)	4 (100)
9.	59	48.0 (7.9)	83	34 (58)	17 (50)	15 (88)	8 (53)	4 (50)
10.	163	55.6 (9.4)	84	23 (14)	8 (35)	7 (88)	4 (57)	4 (100)
Total	1219	50 (9.7)	79	487 (40)	181 (37)	107 (59)	76 (71)	70 (92)

### Interviews and conferences

*Situation before the Breakthrough project*

Participants often described somatic (i.e., of the body) and/or infectious diseases as “only a small part of the job” or perceived them as “extra responsibilities” that frequently got neglected if their regular tasks were too time-consuming. They indicated that awareness of HCV had sometimes peaked during participation in different projects but often faded thereafter. Participants spoke about contributing factors such as “outdated protocols” and “a high workload.” A renewed incitement had been the biannual somatic screening, including possible HCV screening, that was made obligatory by inclusion in the professional guideline in 2012 [18]. These updated guidelines had been part of the motivation to participate in the Breakthrough project for some.

#### Experiences with the Breakthrough project

The Breakthrough project had been an overall good experience for the participants. The interviewees expressed their content about a stimulating team spirit and the inspiring central conferences with high educational value. They learned that their knowledge of HCV had been insufficient prior to the project.

It occurs much more often than you would assume based on symptoms only or than those diagnosed by coincidence.” […] “A lot of clients say: “No, testing is not necessary for me because I do not inject!” Well, we know better by now so we dispute that argument.

Participants specifically praised the motivating feedback they received from the expert team. They indicated that some tempering of their enthusiasm had contributed to setting more realistic goals and that they had actually felt over-demanded during previous projects.

The experts were very helpful. Most of all during the roll-out: “So when you start, what group will you target first and how will you go about this?” Then it quickly became clear we were taking on way too much. This was very insightful.

Essential elements for successful participation in the Breakthrough project constituted full support of both the management and a committed hepatology specialist, as indicated by the different team members. Because teams consisted of specialists and nurses of both addiction care and hepatitis centers, broad support was created within organizations. The close collaboration with the hepatitis treatment center had made it easier to consult with the specialized hepatology nurse about individual clients and to refer them when needed. A further expansion of the multidisciplinary team through the involvement of healthcare partners outside of the addiction care center such as mental health services, sheltered housing, or other outpatient care service providers or even a neighborhood policeman was also positively evaluated.

Two interviewees affirmed that the HCV healthcare pathways had blended in completely with their daily activities after the Breakthrough project. To secure this final integration, several teams had incorporated the HCV healthcare pathway in an overall somatic screening protocol.

I’m proud of what we’ve achieved - we have a hepatitis C pathway. It’s not only about actually having one, […], but mostly that it’s a part of the usual care we provide.

Naturally, all teams also experienced difficulties in the implementation of the pathway such as “changes in staff,” “limited capacity of the hepatitis treatment center for which reason implementation was phased,” and “a shortage in addiction care specialists who were responsible for the somatic screening.” “Registration” was a key limiting factor, both before as well as during the project. For instance, many teams experienced problems in finding the results of previous hepatitis C screenings in patient files. The HAC Breakthrough project members put emphasis on the need and benefits of proper registration.

#### HCV healthcare pathway: best practices and points of improvement

During the central conferences, a wide variety of tips and best practices were summarized that helped to limit drop out of PWID in the HCV cascade of care according to the participants. The main finding is that the nurses—from addiction care and hepatitis center working together—played a key role in making the project a success.

To optimize the *pre-screening phase*, several teams organized training in (motivational) counseling for the addiction care staff in order to inform clients about their risk behavior and to persuade them to get tested. Also, hepatitis nurses were trained on the subject of addiction, so that they would have a better understanding of patients’ behavior. In the *screening phase*, a number of teams had positive experiences with the use of oral swabs for anti-HCV testing. Team members felt that clients were more inclined to have an oral swab test and were more motivated to have further blood tests if the oral swab test came out positive. Other teams opted for immediate blood testing because of the advantage to simultaneously test for HBV or HIV. Some addiction care units facilitated complementary HCV RNA testing in case of a positive anti-HCV test by storing some extra blood. As a solution for the difficult-to-reach clients who are often a “no-show” at the outpatient laboratory service, teams suggested “on-site venipuncture” or “supervised visits of laboratory facilities.” Both solutions however would require extra financial resources for the additional tasks and/or training of the addiction care workers which was not always an option. During the *work-up and treatment phase*, a psychiatric consultation and the evaluation of current birth control measures were found relevant by various teams and this was organized upon indication. Several addiction care units coordinated hospital visits accompanied by the addiction care nurse, outpatient care services, or a sheltered housing supervisor during treatment on the basis of the clients’ specific needs. Naturally, clients preferred a fibroscan over a liver biopsy and a one-stop-shop appointment over multiple hospital visits as was reported by the team members. A number of teams reported positive results on the employment of peer support during HCV therapy. A barrier for the addiction care workers to follow up on the client’s treatment progress at the hepatitis treatment center was that following up on treatment progress is not the main responsibility of the addiction care specialist but that of the hepatology specialist and/or the general practitioner.

## Discussion

To our knowledge, this is the first study to assess the Breakthrough method for a quality improvement project in addiction care in order to contribute to final HCV elimination in PWID. Prior to the HAC Breakthrough project, most of the participating addiction care units had no organized HCV care at all. The HAC Breakthrough project aimed to ensure linkage to care of HCV-infected PWID by introducing comprehensive and clearly defined local HCV healthcare pathways in 80% of all participating addiction care units. After retrospective quality assessment, 83% of all six pathways, submitted by a total of ten teams, were deemed of either “good” or “sufficient” quality. This was deemed an encouraging result of the HAC Breakthrough project, still taking into account that 2 out of 12 teams failed to present their final healthcare pathway and were excluded from the critical appraisal. Considering the excellent referral rate of 92% in the HAC Breakthrough project, it appears that the healthcare pathways have contributed to the realization of the other main project aim, which was to achieve adequate linkage to specialist care. Full support of the management and a committed hepatology specialist were considered essential elements for successful participation in the Breakthrough project. In addition, addiction care and hepatitis center nurses working together made the project a success; they were important in making and implementation of the local pathways.

Uptake of HCV screening seemed to have improved during the HAC Breakthrough project: 6.1% of all registered HCV antibody tests were performed each year during the limited time-span of the two series of the HAC Breakthrough project (2013–2016) compared to 2.1% annually in the 11 years before start of the project (from 2002 until 2012). These results suggest an increased efficiency of the HCV screening process even though not all anti-HCV tests could be accounted for due to missing screening dates. These missing test results probably explain the relatively low HCV test uptake in our project compared to two other Dutch studies in PWID (53–66%) [7, 30]. There was an incremental relative yield in the outcomes of each step of the HCV referral cascade: 40% HCV antibody screening uptake (in all clients at risk), 59% HCV-RNA testing uptake (in all HCV antibody positives), and 92% referral rate (in all HCV-RNA positives). This referral rate in the HAC Breakthrough project is considerably higher in comparison to the large Dutch information campaign in PWID in 2009–2010 (92% vs. 77%) [19]. Best practices that contributed to this accomplishment, according to the teams, included training in motivational counseling, oral swabs for anti-HCV testing, facilitating complementary HCV RNA testing, and supervised hospital visits. Outside of the Netherlands, a number of interventions including facilitated referral and scheduling of specialist appointments have been investigated in a randomized clinical trial setting and successfully improved linkage to care (51–82%) in HCV-infected PWID [31]. Differences in design, setting, and outcome measures however complicate direct comparison between studies.

The treatment completion rate in HCV-RNA positives in this study seemed rather low with 39%; however, the project was predominantly run in the pre-DAA era (DAAs became reimbursed by the healthcare insurance as of November 2015) and patients without advanced fibrosis of cirrhosis often remained in outpatient care until the DAAs became available (i.e., DAA warehousing). Treatment uptake might be improved if evidence-based best practices, such as employment of peer workers [32] *or dedicated local facilitators* [33]*, are more broadly implemented in all participating addiction care units.* The high rate of persistent viremia (42%) in anti-HCV positives underlines the necessity of QIC initiatives, like the HAC Breakthrough project, in order to achieve final micro-elimination in this high-risk group. With referral tackled as shown by this study, the largest gap to be closed in the HCV referral cascade in Dutch PWID appears to be the uptake of HCV antibody screening. An absent risk of (possible) exposure to HCV, as indicated by the patient, was the main argument to abstain from anti-HCV screening during the HAC Breakthrough project. However, self-reported exposure or risk behavior has been described as an unreliable parameter to estimate the true risk of HCV. For instance, several studies report high HCV prevalence (41–66%) among people who use drugs who declared never to have shared needles [34–36]. To sidestep this uncertainty, we advocate a “once-in-a-lifetime screening practice” for the presence of HCV antibodies in PWID without further risk behavior, as an absolute minimum. Other explanations for the high drop-off at the anti-HCV testing uptake included clients repeatedly not showing up for blood testing and incomplete registration.

This study has a few limitations. First of all, a quality assessment of the HCV healthcare pathways was done retrospectively. Even though teams were educated on the content and creation of a healthcare pathway, the specific scoring system to assess the pathways was put together after the end of the project, which may have led to lower final scores of the pathways. On the other hand, it can be argued that the current design of the HAC Breakthrough project is a better reflection of daily practice and that its results are therefore more generalizable. Secondly, even though the number of variables was limited, data collection was challenging in this study with a high proportion of missing values. Specifically, information on follow-up in the hepatitis treatment center was not always available due to lack of the client’s consent and also since following-up on HCV treatment was not considered the main responsibility of the addiction care staff during this project. Consequently, differential attrition across the HCV cascade of care cannot be ruled out and may have biased the results in this study. The registration issues are partly inherent, as indicated by several Breakthrough teams, to outdated and unstructured electronic patient files, which complicated the data extraction and also made it more time-consuming. Moreover, data collection on test results had not been the main focus of the HAC Breakthrough project. This might have led to an underestimation of the absolute numbers of HCV-infected patients who were diagnosed, referred, and completed treatment. As mentioned previously, treatment uptake and completion in PWID are expected to have increased drastically with the current availability of highly efficient DAA therapy. Finally, the number of qualitative in-depth interviews was relatively small but these could fortunately be complemented with the conference summary reports. The findings of this study are applicable to settings with highly accessible HCV and SUD care.

## Conclusion

The HCV in Addiction Care Breakthrough project has brought about good quality HCV healthcare pathways in the majority of the participating addiction care centers and has led to improved collaboration between addiction care and hepatitis treatment centers. Furthermore, it has been very successful in linking HCV-infected PWID to care considering the high referral rate. The design of the HAC Breakthrough project in combination with the best practices could be of use for other addiction care centers that aim to improve local HCV care. Uptake of HCV antibody screening and treatment after referral have been identified as the main gap**s** to be closed in the HCV cascade of referral in order to achieve final micro-elimination in this high-risk group.

## Data Availability

The dataset analyzed during the current study is available from the corresponding author on reasonable request.

## Abbreviations

DAA: Direct-acting antiviral
GGZ: Dutch mental healthcare institutions
HBV: Hepatitis B virus
HCC: Hepatocellular carcinoma
HCV: Hepatitis C virus
HIV: Human immunodeficiency virus
OST: Opioid substitution clinics
PDCA: Plan-do-check-act
PWID: Persons who inject drugs
RIOB: Dutch guidelines on opiate maintenance treatment
SVR: Sustained virological response
